# Non-Contact IOP Estimation Based on Corneal Stress Birefringence: Experimental and Computational Validation

**DOI:** 10.3390/s26113289

**Published:** 2026-05-22

**Authors:** Haoyuan Li, Yinda Li, Zhenhua Guo, Yong Zhang

**Affiliations:** School of Science, Hebei University of Technology, Tianjin 300401, China; haoyuanrli@163.com (H.L.); lyinda1@163.com (Y.L.); zhenhuaguo1@arizona.edu (Z.G.)

**Keywords:** intraocular pressure, optical tonometry, stress birefringence, photoelasticity, non-contact measurement, full-field biomechanics, porcine cornea

## Abstract

Accurate intraocular pressure (IOP) assessment is essential for glaucoma diagnosis and follow-up. Conventional contact tonometry (e.g., Goldmann and rebound devices) remains widely used, but its accuracy is affected by operator dependence, alignment errors, and patient discomfort. We present a non-contact IOP estimation framework based on corneal stress birefringence and full-field fringe inversion. Ex vivo porcine corneas were imaged under controlled pressure loading from 15 to 20 mmHg, and a coupled stress-optic/shell mechanics model was used to generate pressure-indexed synthetic fringe fields for inverse fitting. In the 15–18 mmHg range, more than 75% of the estimates were within plus or minus 1 mmHg of the reference pressure; performance declined at 19–20 mmHg, consistent with a stronger nonlinear biomechanical response and reduced fringe separability. Defect experiments further showed that local stiffness loss caused both near-defect distortion and far-field stress redistribution, supporting the need for full-field rather than point-wise analysis. These results indicate that stress-birefringence imaging is a promising route toward non-contact, region-sensitive IOP assessment.

## 1. Introduction

In glaucoma diagnosis and treatment, intraocular pressure (IOP) is a key clinical criterion. Contact methods include Goldmann and rebound tonometers; however, their accuracy is influenced by the patient’s pain tolerance and by operator-related errors in instrument placement (e.g., aperture position) and applanation conditions [1,2]. Therefore, there is a clear need for a reliable alternative.

Based on photoelasticity and stress birefringence, optical methods can provide a non-destructive means of IOP measurement. Early corneal photoelastic studies confirmed the diagnostic significance of birefringence patterns, and recent reviews have further strengthened this view [3,4,5,6]. However, most existing studies focus on local observables without modeling full-field corneal biomechanics; thus, pressure estimates may lack accuracy across the entire corneal surface [7,8].

This paper integrates full-field fringe projection with a mechanics-based optical forward model to estimate IOP while accounting for both global deformation and defect-related perturbations. Using ex vivo porcine corneas, we show that the method is comparable to other models in the 15–18 mmHg range and can quantify the effect of the defect size on local and global behavior.

**Figure 1:**
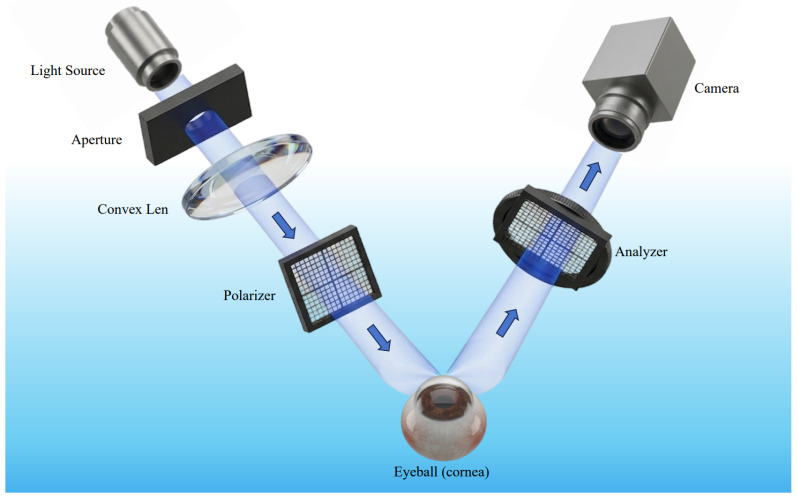
The imaging chain used in this study, including illumination conditioning, polarization control, corneal transmission, and camera capture.

Compared with previous photoelastic IOP studies, the present approach combines full-field fringe information with a mechanics-based optical forward model and evaluates both intact and defected corneas within the same inversion framework. Its main contribution is the integration of stress-optic modeling, multi-feature pressure inversion, and ex vivo cross-validation for region-sensitive IOP estimation. Figure 1 shows the optical path adopted in this study.

The aim of this study is to develop and evaluate a non-contact, full-field IOP estimation method based on corneal stress birefringence. The main findings are that more than 75% of estimates fall within ±1 mmHg in the 15–18 mmHg range, performance decreases at 19–20 mmHg, and defect experiments reveal both near-defect distortion and far-field stress redistribution.

## 2. Methods

### 2.1. Ex Vivo Porcine Cornea Experiment

Isolated porcine eyes provide suitable optical transparency and inflation behavior for validating corneal biomechanics [9,10,11,12,13]. Each specimen was observed in both the intact and defect states under the same loading conditions. The pressure was adjusted between 15.0 and 20.0 mmHg, and six integer pressure levels from 15 to 20 mmHg were chosen as the standard for inverse evaluation.

Paired design aims to achieve within-sample comparisons, reducing intersample variation. Potential ex vivo confounding factors (e.g., water hydration displacement and post-enucleation mechanical preconditioning) were excluded in the experiment; the interpretation focused on relative pressure-related patterns rather than pure stiffness value comparisons. Similar ex vivo control strategies are widely used in corneal optomechanics studies [14,15].

In order to enhance the traceability of data from acquisition to inversion, each pressure level was divided into a short measurement period. Within each block, multiple frames were taken under constant optical conditions and then processed by a uniform quality check; descriptor maps were computed only for frames that passed this test. This reduced the impact of temporary polarization misalignment, short-term vibrations, and local saturation on the final pressure estimation result. Block-based inversion uses descriptors to aggregate information. Thus, it outperforms direct fitting of pixel-level fringes in cases where the local variation of contrast on the corneal surface is uncertain.

**Figure 2:**
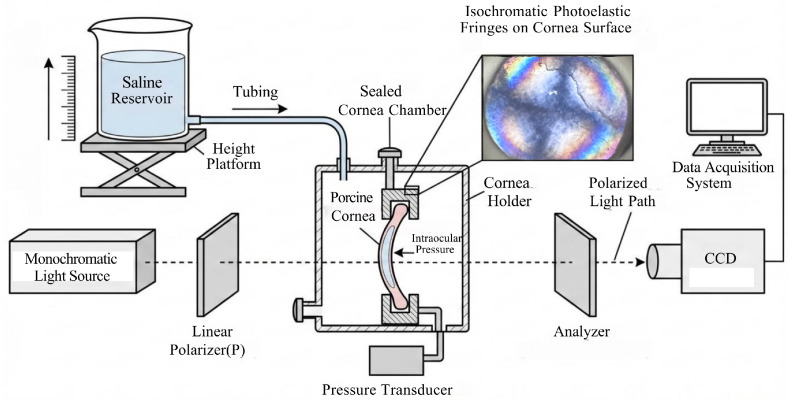
Transmission photoelastic bench used for pressure-tagged acquisition from 15 to 20 mmHg under controlled pressure increments.

In both the intact and defect states, pressure changes were treated as quasi-static processes rather than transients. Therefore, the analysis focused on reproducible spatial structure changes, including fringe crowding, orientation drift, and near-defect deformation. This choice aligned with the assumptions of the current quasi-static model and enabled direct comparison between measured and synthetic fields within the same spatial coordinates. Figure 2 shows the experimental set-up and optical hardware used in this study.

#### 2.1.1. Physical Principle of Stress Birefringence

Within the quasi-steady 15–20 mmHg range, corneal deformation follows a pressure-dependent redistribution on a curved shell. The resulting principal stress difference changes the polarization retardation and therefore alters fringe contrast in transmission microscopy. This mechanism is consistent with classic and recent studies of corneal photoelasticity [3,16,17,18]. To simulate this behavior, birefringence is modeled as the stress-optic response to the principal stress difference, local thickness, and normalized mean stress:

(1) $$\Delta n\left(x,y;P\right)=\left[C_{1}+C_{t}\Theta_{t}\left(x,y\right)+C_{m}{\bar{\sigma }}_{n}\left(x,y;P\right)\right]\Delta \sigma \left(x,y;P\right)+C_{2}\Delta \sigma^{2}\left(x,y;P\right),$$

Here, $\Delta \sigma =\sigma_{1}-\sigma_{2}$ denotes the principal stress difference, $\Theta_{t}=t/t_{0}$ is the normalized thickness, and ${\bar{\sigma }}_{n}=\left(\sigma_{1}+\sigma_{2}\right)/E_{0}$ is the normalized mean stress relative to the reference modulus $E_{0}$. In the low-pressure region (15–18 mmHg), the first-order term $C_{1}+C_{t}\Theta_{t}+C_{m}{\bar{\sigma }}_{n}$ plays a dominant role. At higher pressures (19–20 mmHg), second-order contributions become non-negligible, reflecting nonlinear stress-optic coupling.

For clarity, $C_{1}$ denotes the baseline linear stress-optic coefficient, $C_{t}$ describes the thickness-dependent correction, $C_{m}$ accounts for the mean stress sensitivity, and $C_{2}$ captures the lowest-order nonlinear stress-optic contribution. These coefficients were treated as effective parameters of the complete optical train and were identified once by matching simulated and measured fringe fields from the intact-eye calibration data over the 15–20 mmHg loading range. They were then fixed for all subsequent inverse calculations. The modulus $E_{0}$ serves as the reference tangent elastic modulus used to nondimensionalize the mean stress term and to define the baseline shell stiffness, rather than as a freely varying parameter during inversion.

Optical retardation is converted to a fringe order under monochromatic illumination and fixed path length calibration:

(2) $$\delta_{t}\left(x,y;P\right)=\frac{2\pi t(x,y)}{\lambda }\;\Delta n\left(x,y;P\right),\;N\left(x,y\right)=\frac{\delta_{t}\left(x,y\right)}{2\pi },$$

where t(x,y) is the local thickness, λ is the illumination wavelength, and *N* is the fringe order. Reflection mode interpretation uses the first-order scaling $\delta_{r}\approx 2\delta_{t}$. Dispersion and depolarization effects were included in the effective coefficients without being treated as individual states.

#### 2.1.2. Optical Set-Up and Sample Configuration

The optical bench comprised a monochromator source, a crossed polarizer-analyzer combination, a high-resolution camera, and a hydrostatic pressure loading module [19]. After initial calibration, the light intensity and other acquisition conditions were kept unchanged, except for the pressure. Images were linked to the corresponding pressures and could therefore be compared directly with the simulations.

**Figure 3:**
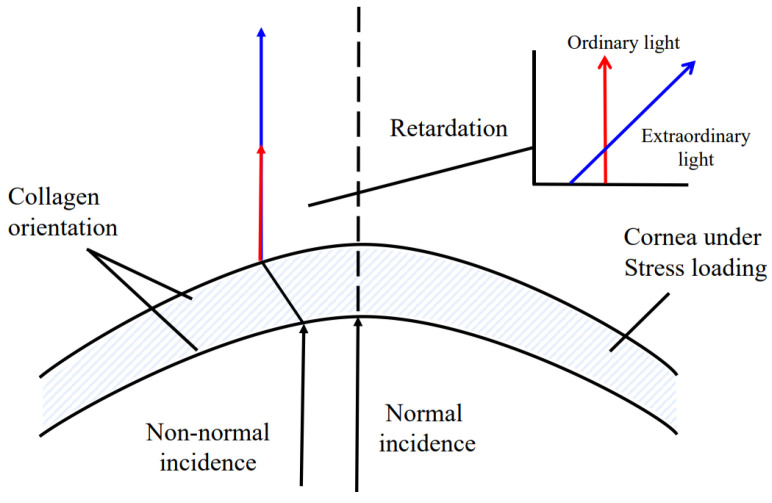
Optical path used for corneal stress-birefringence imaging, showing source illumination, polarization control, transmission through the cornea with stress-induced retardation, analyzer reflection and projection, and final camera recording.

To clarify the birefringence interpretation pathway, Figure 3 summarizes the corneal stress-birefringence optical path used in this study. The incident beam was first conditioned by the polarization control elements and then transmitted through the cornea, where stress-induced retardation accumulated along the optical path, before finally being analyzed before camera recording. In this way, the figure shows how polarization control, corneal transmission, and analyzer reflection jointly determine the observed fringe pattern and why even small polarization state deviations can directly affect decoding accuracy.

Before feature extraction, image registration corrected slight position differences among the scans [20,21,22]. Acquisition and decoding followed the conventional phase step and digital polarization protocol [23,24]. Based on the acquisition log, most frames required only minor adjustment, and the remaining alignment residuals were minimal.

For consistent region-of-interest analysis, the corneal mask was generated for each acquisition block and applied to all descriptors: *N*, ∇N, and *T*. A conservative mask margin was set near the limbus to prevent edge artifacts caused by localized over-excitation of gradient-matching losses. In addition, because the descriptors had quite different scales under stress-induced fringe effects, they were robustly normalized using a percentile-scale method [25].

To reduce non-pressure artifacts, a frame-level quality control gate based on the corneal edge sharpness, color saturation behavior, mask completeness, and polarization-state consistency was used for joint acceptance. Frames that failed the gate were excluded from quantitative inversion while still being retained for visual verification. This separation of quantitative and qualitative use reduces bias transmission from acquisition defects during pressure estimation and is consistent with standard practice in digital photoelastic measurement [19,26,27].

Pressure loading used the saline hydrostatic column method to approximate the aqueous humor pressure difference. The working fluid was physiological saline (0.9% *w*/*v*). The cornea was mounted over a fluid-filled chamber connected to a height-adjustable saline reservoir, and thus the applied load was set by the hydrostatic head acting on the posterior corneal surface. Under this quasi-static condition, the chamber pressure was treated as nearly uniform across the exposed corneal area, while small deviations were expected mainly near the clamped limbal boundary and tubing connection. This means that the measured fringe changes primarily reflect corneal mechanical redistribution under a nominally uniform pressure load rather than strong non-uniform forcing from the chamber itself. The pressure-to-height conversion was computed from the hydrostatic relation P=ρgh, and the corresponding column heights used in this study are listed below.

The conversion was

(3) $$P=\rho gh,\;h=\frac{P_{\mathrm{mmHg}}\times 133.322}{\rho g},$$

where $g=9.80665\;m/s^{2}$. The pressure schedule used in this study was 15.0–20.0 mmHg with 0.5 mmHg increments (11 rounds), and the round-by-round conversion values are listed in Table 1.

### 2.2. Computational Simulation

We implemented a physically constrained Matlab-based framework that integrates geometric definition, pressure application, stress calculation, optical forward projection, and inverse fitting in one workflow [9,11,12,28]. The implementation followed standard digital photoelastic instrumentation and processing techniques. At the same time, we generated six simulation data sets at six pressures matching the experiments for direct model measurement comparison within a common feature space.

At each pressure level, a similar spatial grid and boundary encoding were used to eliminate interlayer differences caused by changes in pressure or material properties rather than mesh inconsistencies. First, the parameter set $\left(R_{x},R_{y},t_{0},\eta ,E_{0},\nu \right)$ was constrained to remain anatomically plausible and was then adjusted within a small, physically interpretable range. Specifically, $R_{x}$ and $R_{y}$ define the two principal radii of curvature, $t_{0}$ is the central thickness, η controls the radial thinning trend, $E_{0}$ is the baseline elastic modulus, and ν is Poisson’s ratio. Geometry-related parameters were obtained from the measured specimen shape before loading, whereas the effective material and optical coefficients were calibrated against the intact-specimen fringe response and then held fixed across pressures and defect simulations. In defective cases, the lesion parameters were adjusted according to the measured deformation without changing the overall geometric assumptions; therefore, all cases remained within the same model family rather than using specimen-specific rules.

#### 2.2.1. Governing Model and Forward Optical Projection

Under quasi-static loading and within the current imaging field of view, we treated the cornea as a shallow elliptical shell with principal radii $R_{x}$ and $R_{y}$. The formulation is not axisymmetric; instead, it allows different curvatures along the two in-plane principal directions while remaining a reduced-order shell model. Rather than using a generic profile, we specifically incorporated the central sag and peripheral thinning by introducing the parameters $t_{0}$ and η:

(4) $$z\left(x,y\right)=h_{c}\left(1-\frac{x^{2}}{R_{x}^{2}}-\frac{y^{2}}{R_{y}^{2}}\right),\;t\left(x,y\right)=t_{0}\left[1-\eta \left(\frac{x^{2}}{R_{x}^{2}}+\frac{y^{2}}{R_{y}^{2}}\right)\right],$$

Here, $h_{c}$ denotes the central sag. At each pressure level *P*, the stress is updated using a coupled membrane–bending shell formulation:

(5) $$\sigma_{x}\left(x,y;P\right)=\frac{PR_{x}}{2t(x,y)}+\frac{6M_{x}\left(x,y;P\right)}{t^{2}\left(x,y\right)},\;\sigma_{y}\left(x,y;P\right)=\frac{PR_{y}}{2t(x,y)}+\frac{6M_{y}\left(x,y;P\right)}{t^{2}\left(x,y\right)},$$

(6) $$M_{x}=D\left(\kappa_{x}-\kappa_{x,0}\right),\;M_{y}=D\left(\kappa_{y}-\kappa_{y,0}\right),\;D=\frac{E\left(x,y\right)t^{3}\left(x,y\right)}{12\left(1-\nu^{2}\right)},$$

Here, $\kappa_{x}$ and $\kappa_{y}$ represent deformed principal curvatures; $\kappa_{x,0}$ and $\kappa_{y,0}$ denote baseline curvatures; and ν is Poisson’s ratio. The coupled model has a stronger pressure-responsive deflection characteristic than the membrane-only approximation, especially under high-stress conditions. In the present study, the shell model is quasi-static and geometrically pressure-updated through the curvature terms, while the constitutive response is kept linear elastic at each load step. Its main assumptions are uniform posterior pressure loading, smooth thickness variation, small local rotations within the imaged central region, and effective representation of a collagen architecture through calibrated coefficients rather than explicit fiber-resolved constitutive laws. Therefore, the model is intended for the controlled ex vivo loading range studied here and is not expected to capture strong viscoelastic relaxation, large-deformation instabilities, or specimen-specific anisotropy beyond what is absorbed into the calibrated parameters [11,28].

Defects were represented as local stiffness reductions rather than as arbitrarily added stress modifications:

(7) $$E_{\mathrm{defect}}\left(x,y\right)=E_{0}\left[1-\alpha_{d}\exp\;\left(-\frac{{\left(x-x_{d}\right)}^{2}}{2a_{d}^{2}}-\frac{{\left(y-y_{d}\right)}^{2}}{2b_{d}^{2}}\right)\right],$$

where $\alpha_{d}$ is the stiffness reduction factor and $\left(x_{d},y_{d},a_{d},b_{d}\right)$ represent the control lesion center and anisotropic spread. These parameters generate focal or incision-like softening and can produce both short-range distortion and long-range fringe displacement features.

**Figure 4:**
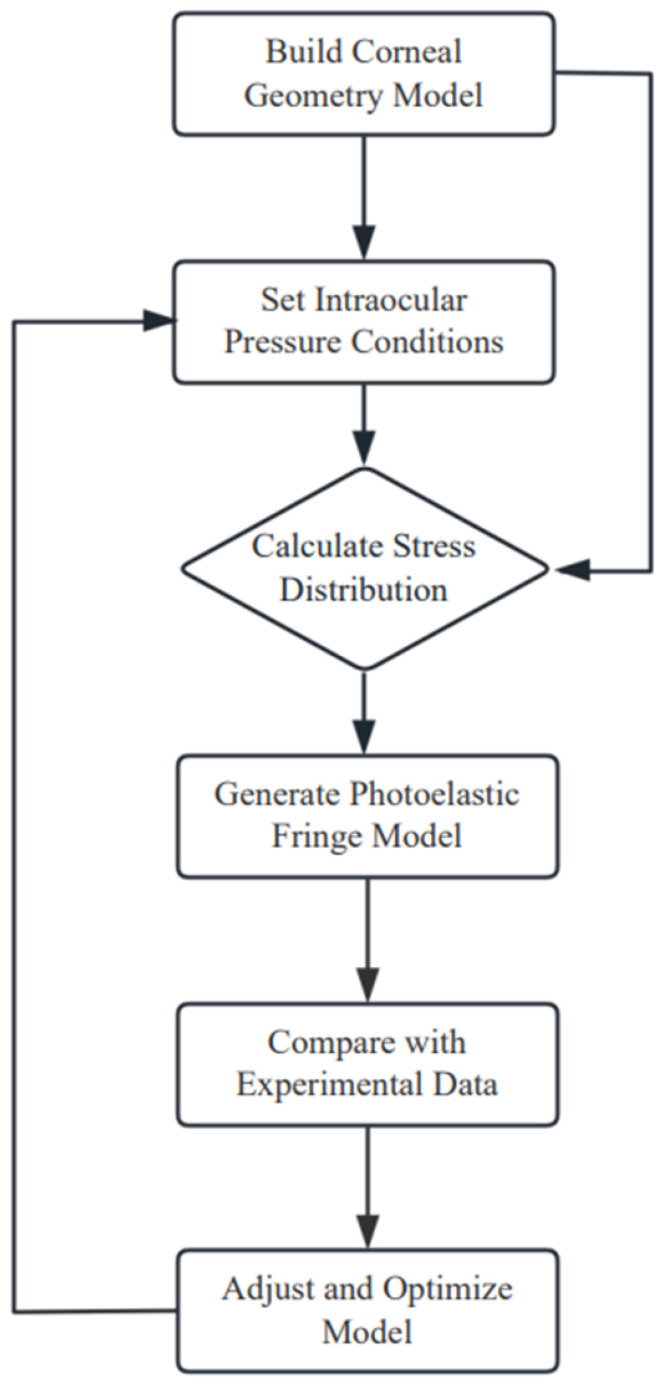
End-to-end computational flow: pressure input, stress field update, stress-optic projection, synthetic fringe generation, and inverse pressure fitting.

Given a certain analyzer orientation and cross-polarization, the stress solution can be transformed into that of birefringence and retardation according to Equations (1) and (2):

(8) $$I_{\mathrm{sim}}\left(x,y;P\right)=I_{0}\sin^{2}\;\left(\frac{\delta_{t}\left(x,y;P\right)}{2}+\phi_{0}\right),$$

where $I_{0}$ is the source intensity and $\phi_{0}$ is the residual phase bias. The forward model yields a pressure-indexed synthetic fringe stack that is compared frame-by-frame with experimental observations. Figure 4 summarizes the end-to-end computational workflow used for pressure estimation.

#### 2.2.2. Fringe Inversion and Pressure Estimation

Pressure inversion uses three complementary full-field descriptors: the fringe order *N*, fringe gradient field ∇N, and texture descriptor *T*. Preprocessing includes Fourier-based normalization and edge-enhanced segmentation to reduce slow illumination drift and preserve weak fringe ridges. The three descriptors jointly improve robustness to contrast variation and local defect perturbation [29,30,31].

Following common digital photoelastic conventions, phase unwrapping and stress parameter stabilization were implemented before model matching [32,33,34]. The IOP was estimated via an exhaustive search over six discrete candidates (15–20 mmHg, 1 mmHg step):

(9) $$\hat{P}=\arg\min_{P\in P}\left[\begin{array}{cc} \; & w_{N}{∥N_{\exp}-N_{\mathrm{sim}}\left(P\right)∥}_{2,\Omega }^{2}\\ \; & +w_{G}{∥\nabla N_{\exp}-\nabla N_{\mathrm{sim}}\left(P\right)∥}_{2,\Omega }^{2}\\ \; & +w_{T}{∥T_{\exp}-T_{\mathrm{sim}}\left(P\right)∥}_{2,\Omega }^{2} \end{array}\right],$$

Here, Ω denotes the valid corneal domain. The weights of the three mismatch terms are $w_{N}$, $w_{G}$, and $w_{T}$. This formulation supports both global field fitting and region-based recognition.

If one term contributes more than about 60% of the total loss, then the optimizer may overemphasize that feature and overfit, similar to the weight sensitivity behavior observed in previous fringe inversion experiments [34,35].

To improve reproducibility, we propose reporting the complete inversion settings, including descriptor-specific normalization, parameter boundaries, and stopping criteria. In actual operation, the weights $w_{N}$, $w_{G}$, and $w_{T}$ should be calibrated on the development set and then kept fixed during assessment. We also suggest recording image quality information such as focus, saturation, and polarization alignment, because these factors directly affect fringe separation and therefore pressure stability [24,26,36]. This recommendation is consistent with quantitative optical metrology practice for improving laboratory-to-laboratory consistency after calibration.

The descriptors’ contribution curves can be monitored across pressure candidates, and residual maps can be computed for each candidate level to better interpret inversion behavior. In this way, global pressure differences can be separated into regions of interest that are easy to interpret. This decomposition helps distinguish genuine biomechanical disagreement from optical deterioration. In particular, persistent residual clusters around the perimeter at higher pressure indicate deficiencies in the model form rather than random camera noise.

#### 2.2.3. Evaluation Metrics and Uncertainty Reporting

To ensure a consistent reproducibility report and provide measures beyond a single summary number, let $P_{i}$ and ${\hat{P}}_{i}$ denote the reference and recovered pressure for sample *i*, respectively, with *n* valid samples. We report the mean absolute error (MAE), root mean square error (RMSE), and within-tolerance rate $R_{\pm 1}$:

(10) $$\mathrm{MAE}=\frac{1}{n}\sum_{i=1}^{n}\left|{\hat{P}}_{i}-P_{i}\right|,\;\mathrm{RMSE}=\sqrt{\frac{1}{n}\sum_{i=1}^{n}{\left({\hat{P}}_{i}-P_{i}\right)}^{2}},$$

(11) $$R_{\pm 1}=\frac{1}{n}\sum_{i=1}^{n}I\left(\left|{\hat{P}}_{i}-P_{i}\right|\leq 1\;\mathrm{mmHg}\right),$$

where I(·) is the indicator function. To characterize the pressure level bias, we additionally report

(12) $$b\left(P_{k}\right)=\frac{1}{n_{k}}\sum_{i\in \Omega_{k}}\left({\hat{P}}_{i}-P_{i}\right),$$

with $\Omega_{k}$ denoting the set of samples at reference level $P_{k}$ and $n_{k}=\left|\Omega_{k}\right|$.

Ex vivo data sets are generally of a moderate size; therefore, uncertainty was quantified by bootstrap confidence intervals obtained by resampling specimens rather than by reporting single values only. After the paired experiment, the resulting inferences remained consistent across specimens and pressure levels. This reporting strategy improves transparency when comparing the low-pressure and high-pressure regions, where residual variance was not uniformly distributed.

In addition, the distributions of the descriptors at each level were tracked through their median, interquartile range, and outliers. This helped determine whether increasing pressure-level errors arose from system-model mismatches or from attenuation of image quality at that level. If the confidence interval and descriptor distribution conflicted, then the result was considered “diagnostically unreliable”, which reduced over-interpretation of individual points. Near 19–20 mmHg, the sample cluster was sparse, and small variations more easily affected phase reconstruction. These additional statistics improved reproducibility and clarifies uncertainty beyond the MAE and RMSE.

## 3. Results

### 3.1. Ex Vivo Porcine Cornea Results

**Figure 5:**
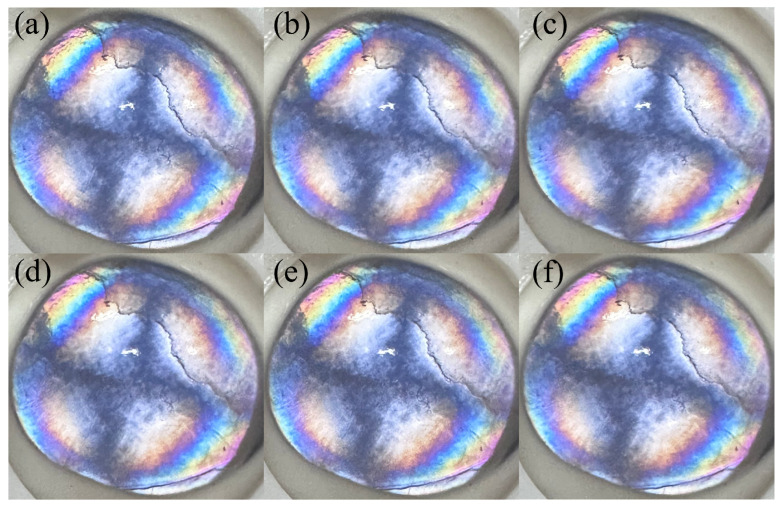
Experimental fringe sequence of intact cornea at six pressure points: (**a**) 15 mmHg; (**b**) 16 mmHg; (**c**) 17 mmHg; (**d**) 18 mmHg; (**e**) 19 mmHg; and (**f**) 20 mmHg, showing progressive crowding as pressure increased.

Fringe spacing decreased continuously as the pressure increased in the intact cornea. As the pressure increased, the peripheral fringe curvature and crowding became progressively stronger (Figure 5) [1,2,8,16,37]. This trend was consistent across samples and was more pronounced at the corneal periphery. These pressure-induced fringe variations indicate that decoding performance depends not only on local contrast but also on how pressure redistributes stress over a curved shell with a varying thickness.

**Figure 6:**
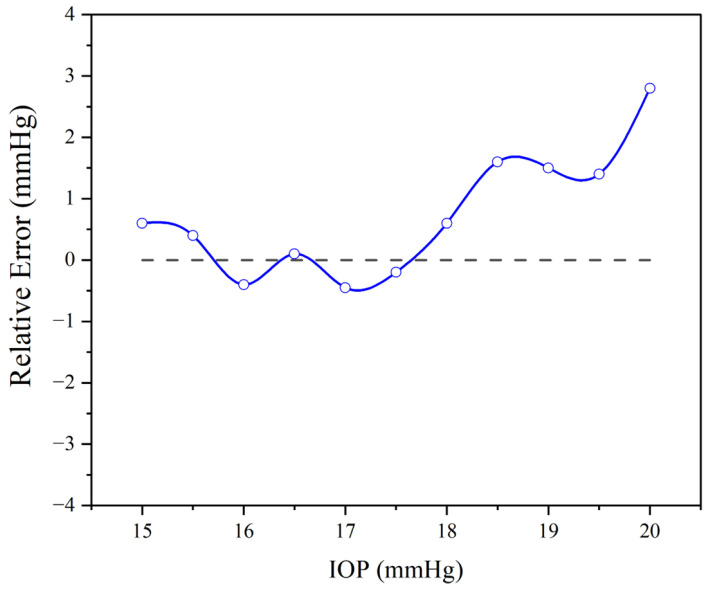
Image-domain relative error versus pressure and imposed pressure versus recovered pressure comparison from inversion.

The recovered pressure versus imposed pressure comparison is shown in Figure 6 [1,2,32,33]. In the 15–18 mmHg range, more than 75% of the estimates were within ±1 mmHg. Accuracy decreased at 19–20 mmHg, where the within ±1 mmHg proportion dropped by approximately 50% relative to the lower-pressure range. The same transition can be seen in the descriptor maps; high-pressure frames had denser fringe arrangements, weaker separation between adjacent bands, and higher sensitivity to small registration errors in the gradient descriptors:

(13) $$\hat{P}=aP+b+\varepsilon ,$$

where *a*, *b*, and ε denote the gain, bias, and residual error, respectively. To interpret the residual structure, we decomposed ε into optical, mechanical, and material components:

(14) $$\varepsilon =\varepsilon_{\mathrm{opt}}+\varepsilon_{\mathrm{mech}}+\varepsilon_{\mathrm{mat}},$$

The local sensitivity term is defined as follows:

(15) $$S_{P}=\frac{\partial N}{\partial P}=\frac{t}{\lambda }\frac{\partial (\Delta n)}{\partial P},$$

Here, *t* and λ are the local thickness and illumination wavelength, respectively [9,10,13,18]. At higher pressures, the fringe overlap and contrast loss increased $\epsilon_{\mathrm{opt}}$; stronger bending and curvature sensitivity increased $\epsilon_{\mathrm{mech}}$; and pressure-dependent effective stiffness variation increased $\epsilon_{\mathrm{mat}}$. Together, these effects explain the wider error range in the high-pressure region and why high-pressure mismatching cannot be fully addressed through image preprocessing alone.

Mechanically, the above evidence shows that pressure dependence primarily results from changes in biomechanical load distribution rather than from pattern recognition alone. Peripheral crowding growth with relatively stable global orientation is consistent with an increasing stress gradient magnitude under preserved structural anisotropy [3,18,38]. As a result, local image-based improvements alone cannot fully restore performance in the 19–20 mmHg range without accounting for pressure-dependent mechanics.

**Figure 7:**
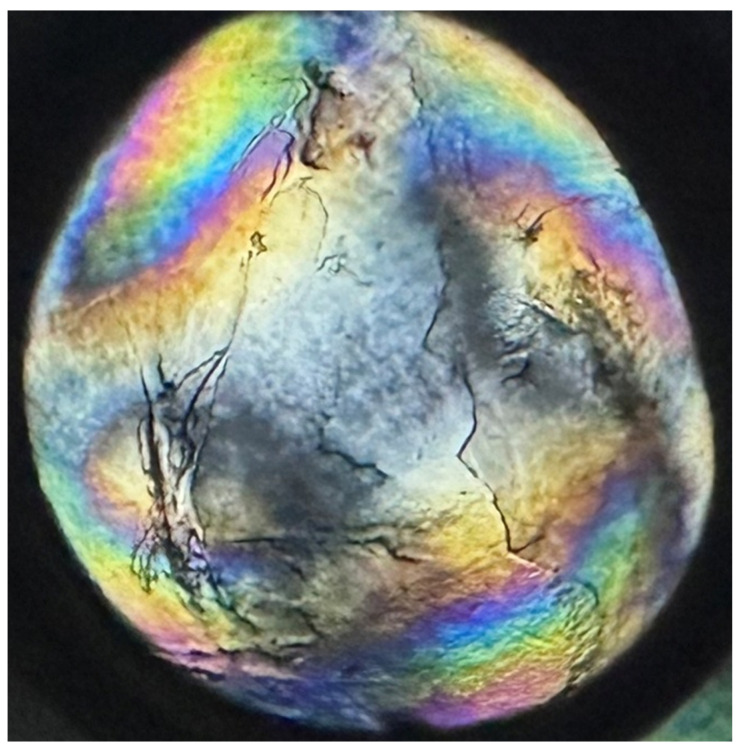
Measured fringe maps before and after defect creation, showing severe distortion near the defect and secondary far-field drift.

In defect experiments, fringe disorder was strongest within approximately 20% of the corneal radius around the lesion and remained detectable in the far field (Figure 7) [8,14,39,40]. This reflects a global load-balancing mechanism rather than a purely local one. Lesion-associated fringe perturbation is not isotropic; the near-defect regions showed abrupt distortion, whereas remote regions exhibited lower-amplitude but spatiotemporally coherent drift. This two-scale behavior motivates a region-anchored inversion strategy, because a single global weight can suppress both lesion-edge artifacts and distant diagnostic details.

Based on the above metrics, the low-to-moderate pressure range showed relatively small measurement errors and stable performance, whereas the 19–20 mmHg range showed a clear deviation trend. The heteroscedastic residuals indicate that fringe overlap, weakened local contrast, and increased bending sensitivity jointly increased the inversion error. Level-wise bias analysis also shows that the high-pressure deviation was systematic; therefore, future model improvements may require pressure-adjusted weights or nonlinear parameter updates.

### 3.2. Computational Results and Cross-Validation

**Figure 8:**
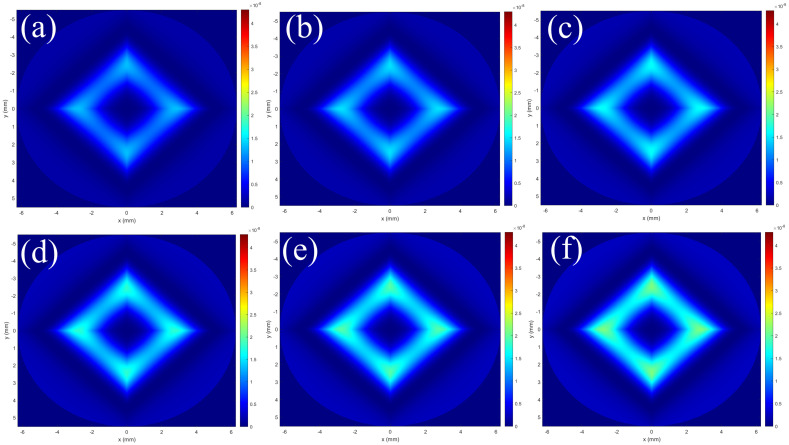
Simulated stress-birefringence intensity maps at the same six pressure points as in the experiment: (**a**) 15 mmHg; (**b**) 16 mmHg; (**c**) 17 mmHg; (**d**) 18 mmHg; (**e**) 19 mmHg; and (**f**) 20 mmHg, enabling one-to-one cross-pressure comparison. The color scale indicates pressure-associated stress-birefringence intensity in the cornea. Darker blue denotes lower intensity and lower pressure-associated stress, whereas lighter cyan-to-yellow denotes higher intensity and a higher stress concentration. The progressive brightening and narrowing of the band with pressure correspond to stronger fringe crowding and weaker separation between adjacent bands, consistent with the experimental observations.

The computations agreed qualitatively with the measurement results. In Figure 8, the color scale represents the simulated stress-birefringence intensity associated with corneal stress redistribution under each imposed IOP level. Here, darker blue regions indicate a lower stress-birefringence intensity and lower pressure-associated stress, whereas lighter cyan-to-yellow regions indicate a higher intensity and higher pressure-associated stress concentration [11,12,24,28]. As the pressure increased from 15 to 20 mmHg, the high-intensity band became brighter and more concentrated, indicating a stronger stress gradient and reduced separation between adjacent fringe bands in the corresponding simulated pattern. This simulated evolution matches the experimental trend in Figure 5, where higher pressures caused more severe fringe crowding while the dominant global alignment remained broadly stable. The comparison at matched pressure levels therefore supports that the model captured the main pressure-dependent redistribution of corneal stress without any additional post-processing parameters. Residual differences were mainly caused by weak local contrast and texture sensitivity; this effect has also been reported in digital photoelastic processing [27]. The partial boundary connection after inversion further confirms that the inversion error was coupled to the fringe separability limits in this region.

**Figure 9:**
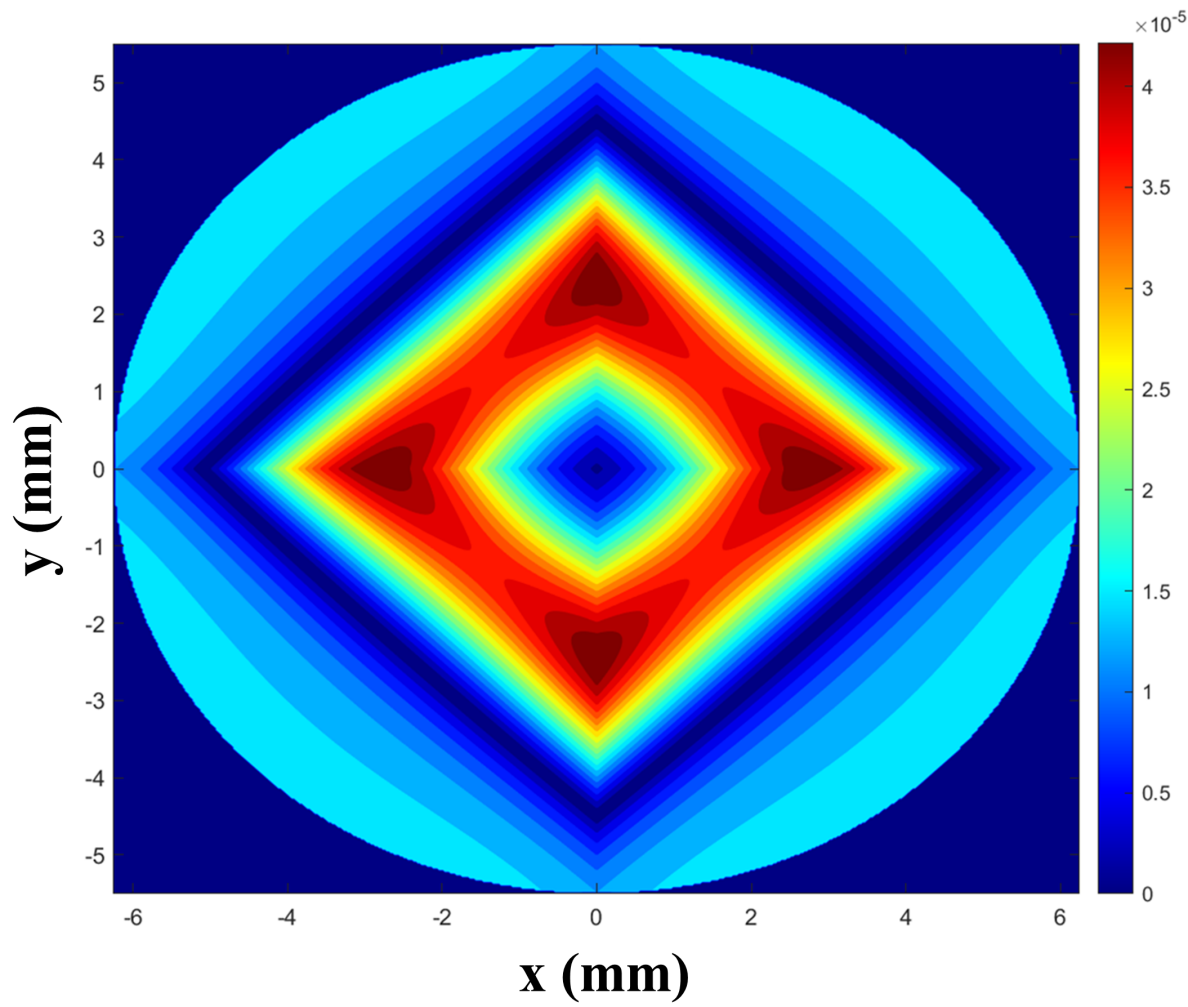
Model-predicted isochromatic order field, with high-order regions aligned to steep stress gradients.

The simulated isochromatic-order map showed high-order bands in regions of steep stress gradients (Figure 9) [7,17,38,41]. More than 75% of these bands overlapped with the experimentally observed fringe crowding at high pressure, indicating that dense fringe areas correspond to mechanically concentrated zones under combined geometric and thickness variation. This agreement supports the use of the coupled membrane–bending approach as a physically meaningful prior in pressure estimation.

**Figure 10:**
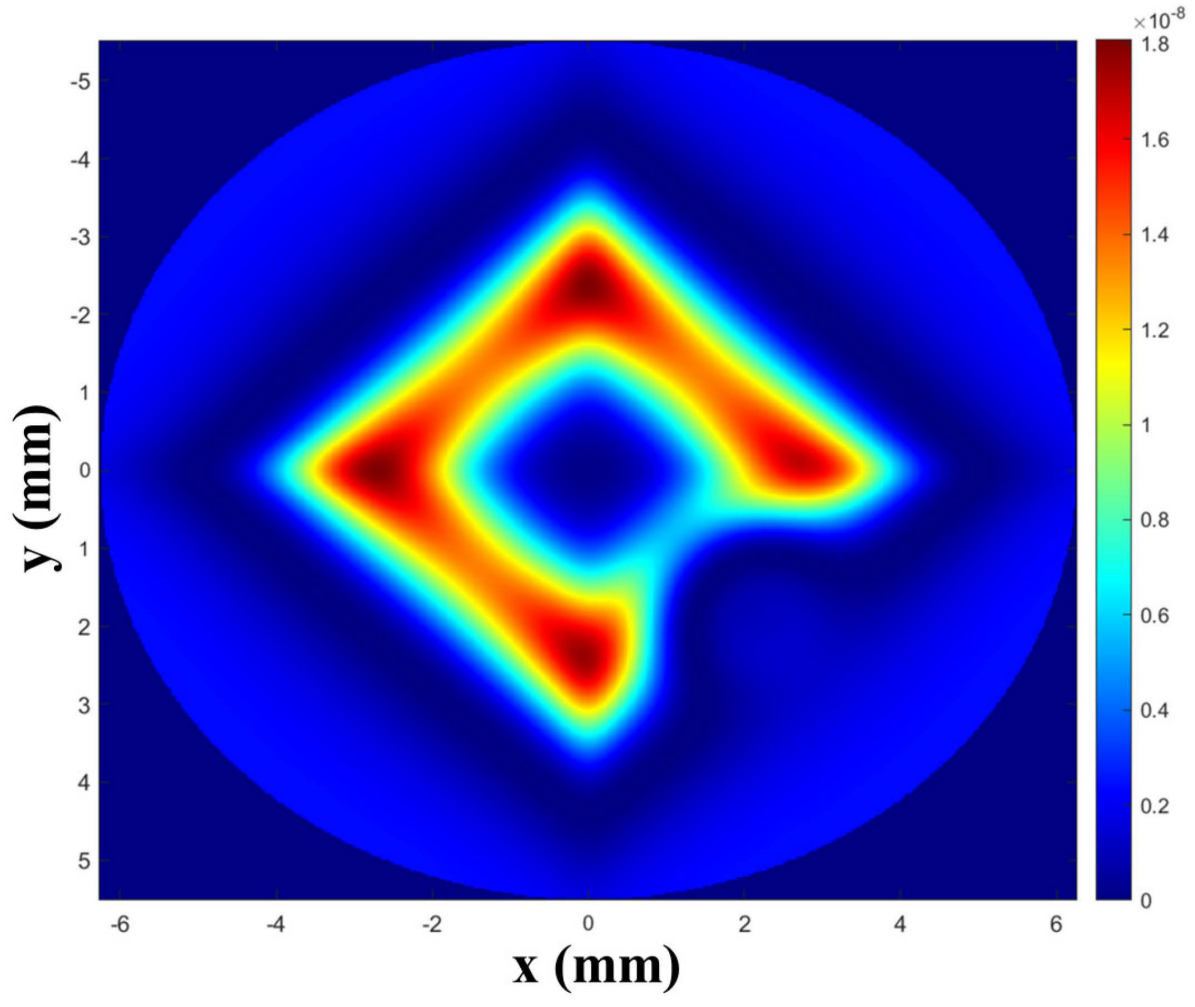
Simulated defect-perturbed stress-birefringence pattern and qualitative consistency with ex vivo imaging.

In defected states, the simulation reproduced strong near-defect stress concentrations with accompanying far-field redistribution (Figure 10) [13,28,39,40].

Lesions with diameters of less than one quarter of the corneal radius were well approximated by stiffness reduction representations, and their main spatial deformation trends were consistent with those experimentally observed. The agreement of the simulated and measured fringe disorder indicates that defect-related pressure-encoding errors are predominantly caused by systematic biomechanical reconfiguration rather than random local optical artifacts. Therefore, this also shows that uniform global calibration fails to adapt well for defected areas, and region-sensitive inversion terms should be employed under such conditions.

In the model, the main 19–20 mmHg offset arose under pressure loading because fringe spacing decreased, bending effects increased, and the response became more sensitive to changes in effective stiffness. The current system captures these phenomena only to the first order through multi-feature inversion and nonlinear stress-optic coupling; therefore, some error remains. Similar behavior has also been observed in digital photoelastic tests near the threshold of stable phase reconstruction [32,34,35].

Cross-validation further demonstrates the applicability of the method under realistic conditions. Conventional tonometric methods can measure pressure accurately, but they do not directly resolve regional biomechanical variation. Full-field stress-birefringence imaging provides additional spatial information on stress distribution, defect-induced perturbation, and biomechanical asymmetry [6,8,12,15,42]. Therefore, a practical near-term deployment path is a hybrid workflow; tonometry anchors the baseline IOP, while birefringence inversion contributes regional biomechanical context for interpretation.

At this time, it is still a controlled ex vivo proof of concept. Hydration dynamics, temperature drift, and boundary-condition variability in vitro can all affect the optical contrast and effective stiffness. Additionally, the current simulation is a quasi-steady-state model that lacks viscoelastic relaxation effects and time-variant collagen reorganization phenomena. To move toward in vivo translation, future studies should include subject-specific geometry and material priors, uncertainty-aware pressure outputs, and prospective robustness tests under blink, motion, and tear-film perturbations [4,5,6,11,42]. These measures are needed to transform the current laboratory decoding process into a form that can be tested clinically.

Another implication of the cross-validation result is that quality control should be integrated into the clinical inference interface rather than treated as a separate offline preprocessing task. A deployment-oriented system should report confidence together with $\hat{P}$, indicate whether residuals are concentrated in lesion-adjacent regions, and flag frames that violate the polarization or contrast assumptions used by the forward model. Such a quality-aware report can detect overconfidence in decision making under uncalibrated measurement conditions and can guide reacquisition during routine inspections. Therefore, the system should be regarded as an integrated measurement platform for jointly estimating pressure and data reliability rather than as a single-output regression tool.

## 4. Conclusions

This study presented a non-contact IOP estimation method that combines ex vivo stress-birefringence imaging with mechanics-constrained reconstruction over the 15–20 mmHg range. The pipeline includes stress-optic coupling, membrane–bending shell mechanics, defect-aware stiffness perturbation, and multi-feature full-field fitting. Most estimates fell within ±1 mmHg of the reference value; however, significant errors remained above 20 mmHg because nonlinear high-pressure responses are not yet fully controlled. Defects revealed global load redistribution, which highlights the advantage of full-field optical inspection over single-point scanning. Given the current ex vivo and quasi-static design, direct clinical application should be postponed; next-stage research should focus on in vivo verification, parameter specification, and uncertainty quantification across the optical, mechanical, and material submodels.

## Figures and Tables

**Table 1:** Pressure schedule in Section 2.1.2 and the corresponding physiological saline column heights used in this study.

Pressure (mmHg)	Pressure (Pa)	Saline Column Height (cm)
15.0	1999.84	20.293
15.5	2066.50	20.970
16.0	2133.16	21.646
16.5	2199.82	22.323
17.0	2266.48	22.999
17.5	2333.14	23.675
18.0	2399.80	24.352
18.5	2466.46	25.028
19.0	2533.13	25.705
19.5	2599.79	26.381
20.0	2666.45	27.058

## Data Availability

Pressure labels, processed fringe maps, and inversion outputs can be shared by the corresponding author for academic use after a reasonable request.
